# Supplementary material for: Assessing Delivery of Selected Public Health Operations via Essential Public Health Operation Framework
Source: Int J Environ Res Public Health. 2020 Sep 3;17(17):6435. doi: 10.3390/ijerph17176435 (PMC7503537; doi:10.3390/ijerph17176435)
Supplement: Supplementary file 1 [file ijerph-17-06435-s001.pdf]

University of Southern Denmark

The questionnaire is based on National Longitudinal Survey of Public Health System Instrument for USA developed by Glen Mays University of Kentucky

---

Name of Health Department

______

Job title/profession

______

Name

______

E-mail

______

How many years have you worked in Kolding municipality?

(1) ❑ 1-12 months

(2) ❑ 1-5

(4) ❑ 5-10

(3) ❑ 10+

EPHO 4

1) In the past 3 years in your municipality, has a community needs assessment process been conducted that systematically describes the health status of the municipality?

(1) ❑ Yes

(2) ❑ No

(3) ❑ Comments _______

If YES: What other types of organizations are involved in performing this activity in your jurisdiction? (mark all that apply)

(1) ❑ Kolding Municipality

(2) ❑ The Danish Health Authority

(3) ❑ The Regions

(4) ❑ The Region of Southern Denmark

(5) ❑ The Danish Working Environment (WEA)

(6) ❑ The Danish Ministry of Environment and Food

(7) ❑ Universities

(8) ❑ National Institute of Public Health (SIF)

(9) ❑ Statens Serum Institute (SSI)

(10) ❑ Health Centers

(11) ❑ Hospitals

(12) ❑ Physician Practices

(13) ❑ Health insurers

(14) ❑ Other state gov't agency

(15) ❑ Other non-profits

(16) ❑ None

(17) ❑ Other - specify _____

How much is the budget for this service approximately?

____

____

Who finances this activity or service?

____

____

EPHO 4.

2) In the past 3 years in your municipality, has a survey of the population for behavioral risk factors (fx. smoking habits, alcohol consumption) been conducted?

(1) ❑ Yes

(2) ❑ No

(3) ❑ Comments _______

If YES: What other types of organizations are involved in performing this activity in your jurisdiction? (mark all that apply)

(1) ❑ Kolding Municipality

(2) ❑ The Danish Health Authority

(3) ❑ The Regions

(4) ❑ The Region of Southern Denmark

(5) ❑ The Danish Working Environment (WEA)

(6) ❑ The Danish Ministry of Environment and Food

(7) ❑ Universities

(8) ❑ National Institute of Public Health (SIF)

(9) ❑ Statens Serum Institute (SSI)

(10) ❑ Health Centers

(11) ❑ Hospitals

(12) ❑ Physician Practices

(13) ❑ Health insurers

(14) ❑ Other state gov't agency

(15) ❑ Other non-profits

(16) ❑ None

(17) ❑ Other - specify _____

How much is the budget for this service approximately?

____

____

Who finances this activity or service?

____

____

EPHO 4.

3) What areas of effort does your municipality have within health promotion and prevention?

- ❑ Diet / nutrition
- ❑ Smoking
- ❑ Alcohol
- ❑ Physical Activity
- ❑ Chronic Obstructive Pulmonary Disease (COPD)
- ❑ Mental health
- ❑ Drug abuse
- ❑ CVD
- ❑ Diabetes
- ❑ Other - specify _____

EPHO 3.

4) In your municipality, are timely investigations of adverse health events conducted on an ongoing basis, including communicable disease outbreaks and environmental health hazards?

(1) ❑ Yes

(2) ❑ No

(3) ❑ Comments _______

If YES: What other types of organizations are involved in performing this activity in your jurisdiction? (mark all that apply)

(1) ❑ Kolding Municipality

(2) ❑ The Danish Health Authority

(3) ❑ The Regions

(4) ❑ The Region of Southern Denmark

(5) ❑ The Danish Working Environment (WEA)

(6) ❑ The Danish Ministry of Environment and Food

(7) ❑ Universities

(8) ❑ National Institute of Public Health (SIF)

(9) ❑ Statens Serum Institute (SSI)

(10) ❑ Health Centers

(11) ❑ Hospitals

(12) ❑ Physician Practices

(13) ❑ Health insurers

(14) ❑ Other state gov't agency

(15) ❑ Other non-profits

(16) ❑ None

(17) ❑ Other - specify _____

How much is the budget for this service approximately?

____

____

Who finances this activity or service?

____

____

EPHO 3.

5) In your municipality, are timely investigations of adverse health events conducted on

an ongoing basis, including occupational health risks (illnesses and injuries in the workplace)?

(1) ❑ Yes

(2) ❑ No

(3) ❑ Comments _______

If YES: What other types of organizations are involved in performing this activity in your jurisdiction? (mark all that apply)

(1) ❑ Kolding Municipality

(2) ❑ The Danish Health Authority

(3) ❑ The Regions

(4) ❑ The Region of Southern Denmark

(5) ❑ The Danish Working Environment (WEA)

(6) ❑ The Danish Ministry of Environment and Food

(7) ❑ Universities

(8) ❑ National Institute of Public Health (SIF)

(9) ❑ Statens Serum Institute (SSI)

(10) ❑ Health Centers

(11) ❑ Hospitals

(12) ❑ Physician Practices

(13) ❑ Health insurers

(14) ❑ Other state gov't agency

(15) ❑ Other non-profits

(16) ❑ None

(17) ❑ Other - specify _____

How much is the budget for this service approximately?

____

____

Who finances this activity or service?

____

____

EPHO 5.

6) In your municipality, are the necessary laboratory services (fx. hospitals, physicians' practices) available to support investigations of adverse health events and meet routine diagnostic and surveillance needs for your jurisdiction?

(1) ❑ Yes

(2) ❑ No

(3) ❑ Comments _______

If YES: What other types of organizations are involved in performing this activity in your jurisdiction? (mark all that apply)

(1) ❑ Kolding Municipality

(2) ❑ The Danish Health Authority

(3) ❑ The Regions

(4) ❑ The Region of Southern Denmark

(5) ❑ The Danish Working Environment (WEA)

(6) ❑ The Danish Ministry of Environment and Food

(7) ❑ Universities

(8) ❑ National Institute of Public Health (SIF)

(9) ❑ Statens Serum Institute (SSI)

(10) ❑ Health Centers

(11) ❑ Hospitals

(12) ❑ Physician Practices

(13) ❑ Health insurers

(14) ❑ Other state gov't agency

(15) ❑ Other non-profits

(16) ❑ None

(17) ❑ Other - specify _____

How much is the budget for this service approximately?

____

____

Who finances this activity or service?

____

____

EPHO 4

7) In the past 3 years in your jurisdiction, has an analysis been completed of the determinants of and contributing factors to priority health needs and the population groups most effected?

(1) ❑ Yes

(2) ❑ No

(3) ❑ Comments _______

If YES: What other types of organizations are involved in performing this activity in your jurisdiction? (mark all that apply)

(1) ❑ Kolding Municipality

(2) ❑ The Danish Health Authority

(3) ❑ The Regions

(4) ❑ The Region of Southern Denmark

(5) ❑ The Danish Working Environment (WEA)

(6) ❑ The Danish Ministry of Environment and Food

(7) ❑ Universities

(8) ❑ National Institute of Public Health (SIF)

(9) ❑ Statens Serum Institute (SSI)

(10) ❑ Health Centers

(11) ❑ Hospitals

(12) ❑ Physician Practices

(13) ❑ Health insurers

(14) ❑ Other state gov’t agency

(15) ❑ Other non-profits

(16) ❑ None

(17) ❑ Other - specify _____

How much is the budget for this service approximately?

____

____

Who finances this activity or service?

____

____

EPHO 5.

8) In your municipality, is there a collaboration with other health-related organizations with regard to health promotion and disease prevention initiatives (e.g. screening programs, vaccination programs)?

(1) ❑ Yes

(2) ❑ No

(3) ❑ Comments _______

If YES: What other types of organizations are involved in performing this activity in your jurisdiction? (mark all that apply)

(1) ❑ Kolding Municipality

(2) ❑ The Danish Health Authority

(3) ❑ The Regions

(4) ❑ The Region of Southern Denmark

(5) ❑ The Danish Working Environment (WEA)

(6) ❑ The Danish Ministry of Environment and Food

(7) ❑ Universities

(8) ❑ National Institute of Public Health (SIF)

(9) ❑ Statens Serum Institute (SSI)

(10) ❑ Health Centers

(11) ❑ Hospitals

(12) ❑ Physician Practices

(13) ❑ Health insurers

(14) ❑ Other state gov't agency

(15) ❑ Other non-profits

(16) ❑ None

(17) ❑ Other - specify _____

How much is the budget for this service approximately?

____

____

Who finances this activity or service?

____

____

EPHO 3, 4, 5.

9) In your municipality, is there a network of support and communication relationships with other health-related organizations about the health problems in the community?

(1) ❑ Yes

(2) ❑ No

(3) ❑ Comments _______

If YES: What other types of organizations are involved in performing this activity in your jurisdiction? (mark all that apply)

(1) ❑ Kolding Municipality

(2) ❑ The Danish Health Authority

(3) ❑ The Regions

(4) ❑ The Region of Southern Denmark

(5) ❑ The Danish Working Environment (WEA)

(6) ❑ The Danish Ministry of Environment and Food

(7) ❑ Universities

(8) ❑ National Institute of Public Health (SIF)

(9) ❑ Statens Serum Institute (SSI)

(10) ❑ Health Centers

(11) ❑ Hospitals

(12) ❑ Physician Practices

(13) ❑ Health insurers

(14) ❑ Other state gov't agency

(15) ❑ Other non-profits

(16) ❑ None

(17) ❑ Other - specify _____

How much is the budget for this service approximately?

____

____

Who finances this activity or service?

____

____

EPHO 4 and 5.

10) Does the municipality coordinate action plans with other health-related organizations?

(1) ❑ Yes

(2) ❑ No

(3) ❑ Comments _______

If YES: What other types of organizations are involved in performing this activity in your jurisdiction? (mark all that apply)

(1) ❑ Kolding Municipality

(2) ❑ The Danish Health Authority

(3) ❑ The Regions

(4) ❑ The Region of Southern Denmark

(5) ❑ The Danish Working Environment (WEA)

(6) ❑ The Danish Ministry of Environment and Food

(7) ❑ Universities

(8) ❑ National Institute of Public Health (SIF)

(9) ❑ Statens Serum Institute (SSI)

(10) ❑ Health Centers

(11) ❑ Hospitals

(12) ❑ Physician Practices

(13) ❑ Health insurers

(14) ❑ Other state gov't agency

(15) ❑ Other non-profits

(16) ❑ None

(17) ❑ Other - specify _____

How much is the budget for this service approximately?

____

____

Who finances this activity or service?

____

____

EPHO 4.

11) In the past 3 years in your municiplaity, has a community health action plan been developed with community participation to address community health needs?

(1) ❑ Yes

(2) ❑ No

(3) ❑ Comments _____

If YES: Overall, how well is this activity performed within your municipality?

(1) ❑ Poor - meets none of the need for this activity

(2) ❑ Moderate - meets about half of the need

(3) ❑ Good - meets most of the need for this activity

(4) ❑ Excellent – fully meets need for this activity

If YES: What other types of organizations are involved in performing this activity in your jurisdiction? (mark all that apply)

(1) ❑ Kolding Municipality

(2) ❑ The Danish Health Authority

(3) ❑ The Regions

(4) ❑ The Region of Southern Denmark

(5) ❑ The Danish Working Environment (WEA)

(6) ❑ The Danish Ministry of Environment and Food

(7) ❑ Universities

(8) ❑ National Institute of Public Health (SIF)

(9) ❑ Statens Serum Institute (SSI)

(10) ❑ Health Centers

(11) ❑ Hospitals

(12) ❑ Physician Practices

(13) ❑ Health insurers

(14) ❑ Other state gov't agency

(15) ❑ Other non-profits

(16) ❑ None

(17) ❑ Other - specify _____

How much is the budget for this service approximately?

____

____

Who finances this activity or service?

____

____

EPHO 4.

12) In the past 3 years in your municipality, have plans been developed to allocate resources in a manner consistent with priorities established from a community health needs assessment?

(1) ❑ Yes

(2) ❑ No

(3) ❑ Comments _______

If YES: What other types of organizations are involved in performing this activity in your jurisdiction? (mark all that apply)

(1) ❑ Kolding Municipality

(2) ❑ The Danish Health Authority

(3) ❑ The Regions

(4) ❑ The Region of Southern Denmark

(5) ❑ The Danish Working Environment (WEA)

(6) ❑ The Danish Ministry of Environment and Food

(7) ❑ Universities

(8) ❑ National Institute of Public Health (SIF)

(9) ❑ Statens Serum Institute (SSI)

(10) ❑ Health Centers

(11) ❑ Hospitals

(12) ❑ Physician Practices

(13) ❑ Health insurers

(14) ❑ Other state gov't agency

(15) ❑ Other non-profits

(16) ❑ None

(17) ❑ Other - specify _____

How much is the budget for this service approximately?

____

____

Who finances this activity or service?

____

____

EPHO 3, 4, and 5.

13) In your municipality, is existing evidence (epidemiological studies, etc.) used as a basis for decision-making regarding prioritization of focus areas?

(1) ❑ Yes

(2) ❑ No

(3) ❑ Comments _____

If YES: Overall, how well is this activity performed within your municipality?

(1) ❑ Poor - meets none of the need for this activity

(2) ❑ Moderate - meets about half of the need

(3) ❑ Good - meets most of the need for this activity

(4) ❑ Excellent – fully meets need for this activity

If YES: What other types of organizations are involved in performing this activity in your jurisdiction? (mark all that apply)

(1) ❑ Kolding Municipality

(2) ❑ The Danish Health Authority

(3) ❑ The Regions

(4) ❑ The Region of Southern Denmark

(5) ❑ The Danish Working Environment (WEA)

(6) ❑ The Danish Ministry of Environment and Food

(7) ❑ Universities

(8) ❑ National Institute of Public Health (SIF)

(9) ❑ Statens Serum Institute (SSI)

(10) ❑ Health Centers

(11) ❑ Hospitals

(12) ❑ Physician Practices

(13) ❑ Health insurers

(14) ❑ Other state gov't agency

(15) ❑ Other non-profits

(16) ❑ None

(17) ❑ Other - specify _____

EPHO 4.

14) In the past 3 years in your municipality, have there been regular evaluations of the effects of public health services on community health status?

(1) ❑ Yes

(2) ❑ No

(3) ❑ Comments _______

If YES: What other types of organizations are involved in performing this activity in your jurisdiction? (mark all that apply)

(1) ❑ Kolding Municipality

(2) ❑ The Danish Health Authority

(3) ❑ The Regions

(4) ❑ The Region of Southern Denmark

(5) ❑ The Danish Working Environment (WEA)

(6) ❑ The Danish Ministry of Environment and Food

(7) ❑ Universities

(8) ❑ National Institute of Public Health (SIF)

(9) ❑ Statens Serum Institute (SSI)

(10) ❑ Health Centers

(11) ❑ Hospitals

(12) ❑ Physician Practices

(13) ❑ Health insurers

(14) ❑ Other state gov't agency

(15) ❑ Other non-profits

(16) ❑ None

(17) ❑ Other - specify _____

How much is the budget for this service approximately?

____

____

Who finances this activity or service?

____

____

EPHO 5:

15) In the past 3 years in your municipality, have professionally recognized process and outcome measures been used to monitor public health programs and to redirect resources as appropriate?

(1) ❑ Yes

(2) ❑ No

(3) ❑ Comments _______

If YES: What other types of organizations are involved in performing this activity in your jurisdiction? (mark all that apply)

(1) ❑ Kolding Municipality

(2) ❑ The Danish Health Authority

(3) ❑ The Regions

(4) ❑ The Region of Southern Denmark

(5) ❑ The Danish Working Environment (WEA)

(6) ❑ The Danish Ministry of Environment and Food

(7) ❑ Universities

(8) ❑ National Institute of Public Health (SIF)

(9) ❑ Statens Serum Institute (SSI)

(10) ❑ Health Centers

(11) ❑ Hospitals

(12) ❑ Physician Practices

(13) ❑ Health insurers

(14) ❑ Other state gov't agency

(15) ❑ Other non-profits

(16) ❑ None

(17) ❑ Other - specify _____

How much is the budget for this service approximately?

____

____

Who finances this activity or service?

____

____

EPHO 5.

16) In the past 3 years in your municipality, has the public regularly received information about current health status, health care needs, health behaviors, and health care policy issues?

(1) ❑ Yes

(2) ❑ No

(3) ❑ Comments _______

If YES: What other types of organizations are involved in performing this activity in your jurisdiction? (mark all that apply)

(1) ❑ Kolding Municipality

(2) ❑ The Danish Health Authority

(3) ❑ The Regions

(4) ❑ The Region of Southern Denmark

(5) ❑ The Danish Working Environment (WEA)

(6) ❑ The Danish Ministry of Environment and Food

(7) ❑ Universities

(8) ❑ National Institute of Public Health (SIF)

(9) ❑ Statens Serum Institute (SSI)

(10) ❑ Health Centers

(11) ❑ Hospitals

(12) ❑ Physician Practices

(13) ❑ Health insurers

(14) ❑ Other state gov't agency

(15) ❑ Other non-profits

(16) ❑ None

(17) ❑ Other - specify _____

How much is the budget for this service approximately?

____

____

Who finances this activity or service?

____

____

EPHO 5 and 4.

17) Select the top 3 methods that the municipality use to educate people about the public health services available to them.

(11) ❑ Facebook

(1) ❑ Twitter

(2) ❑ Instagram

(3) ❑ Press release

(4) ❑ Purchase media

(5) ❑ News Paper

(6) ❑ Meeting with healthcare provider

(7) ❑ Brochures

(8) ❑ E-mail

(9) ❑ Website

(10) ❑ Posters/ Flyers

(12) ❑ None

(13) ❑ Other - specify _____
